# Buruli Ulcer: Treatment Challenges at Three Centres in Ghana

**DOI:** 10.1155/2012/371915

**Published:** 2012-11-29

**Authors:** Pius Agbenorku, Isaac K. Donwi, Pawson Kuadzi, Paul Saunderson

**Affiliations:** ^1^Reconstructive Plastic Surgery and Burns Unit, Komfo Anokye Teaching Hospital School of Medical Sciences, Kwame Nkrumah University of Science and Technology, Kumasi, Ghana; ^2^Department of Mathematics, College of Science, Kwame Nkrumah University of Science and Technology, Kumasi, Ghana; ^3^American Leprosy Missions, 1 Alm Way, Greenville, SC 29601, USA

## Abstract

*Aims.* This retrospective study was to identify some challenges in the treatment of Buruli ulcer (BU) and present a proposed treatment regime. *Materials and Methods.* Information from patients medical records, hospital database, and follow-up findings on BU treatment procedures from 1994 to 1998 and from 2004 to 2007 at three research sites in Ghana were reviewed to determine the treatment challenges encountered. Data needed were recorded and analyzed, and results presented using SPSS version 17.0. *Results.* A total of 489 BU patients information was selected for the study. A majority (56.90%, *n* = 278) of the patients were children (0–14 years), with a mean age of 12.8 years. Significant challenges in BU treatment in Ghana identified included sequelae (*P* = 0.041 ), delayed treatment (*P* = 0.012 ), and high treatment cost (*P* = 0.044 ). Duration of hospital stay was clearly correlated with the time spent at home prior to admission; spearman's rank correlation coefficient was 0.72 (95% CI 0.42–0.87). *Conclusion.* Delays in seeking treatment among BU patients were the main factor which resulted in most of the other factors contributing to the challenges in treatment. A combination of psychosocial and biomedical approach was proposed as holistic method to alleviate the challenges in BU treatment.

## 1. Introduction

Buruli ulcer (BU), the *Mycobacterium ulcerans* skin ulcer, also known as Bairnsdale ulcer, Daintree ulcer, Mossman ulcer, Kumusi ulcer, Tontokrom ulcer, and Searles' ulcer, is a chronic, indolent, and necrotizing disease of the skin and soft tissue caused by *Mycobacterium ulcerans (MU)*, which usually begins as a painless nodule or papule and may progress to massive skin ulceration [1–3]. If untreated, BU may lead to extensive soft-tissue destruction, with inflammation and subsequent complete tissue necrosis extending to deep fascia and the bone. The parts of the body mostly affected are the extremities, especially the lower ones. Subsequent complications may include contracture deformities, leading to loss or limitations in function and even amputations. 

BU disease is assuming public health importance in many countries, prompting the establishment of the “Global Buruli Ulcer Initiative” by the World Health Organization (WHO) in early 2008 [1].* MU* infection was first reported in Bairnsdale, southeast Australia in 1948 [4] and later named Buruli ulcer in Uganda [5]. *MU* is the third most common mycobacterial infection worldwide, after tuberculosis and leprosy [6, 7]. BU mainly affects individuals in humid, rural, tropical regions with limited access to medical care. BU frequently occurs near water bodies—slow flowing rivers, ponds, swamps, and lakes; cases have also occurred following flooding. 

Activities that take place near water bodies, such as farming, are risk factors, and wearing protective clothing appears to reduce the risk of the disease. The reasons for the growing spread of BU remain unclear. All ages and sexes are affected, but most patients are among children under 15 years [8]. In general, there is no difference in the infection rate among males and females. The disease can affect any part of the body, but in about 90% of cases the lesions are on the limbs, with nearly 60% of all lesions on the lower limbs [9]. Unlike tuberculosis, there is no evidence to suggest that infection with the human immunodeficiency virus predisposes individuals to BU infection. There is also no evidence that the disease can be transmitted from person to person. There is little seasonal variation in the incidence of the disease [10–12]. 

It has been reported in about 30 tropical countries, with the greatest frequency in Africa, particularly in the west African countries of Côte d'Ivoire, Ghana, and Bénin (20 to 158 cases per 100,000) [8–14], where the peak age group is 5 to 15 years; BU can affect any age group [6, 7]. The goal of this retrospective study is to identify challenges in the treatment of BU in different areas of Ghana and present a proposed treatment regime.

## 2. Materials and Methods

### 2.1. Treatment Centers

The Komfo Anokye Teaching Hospital (KATH), located in Kumasi, is the second-largest hospital in Ghana and the only tertiary health institution in the middle belt of the country. The hospital currently has 1000 beds, up from the initial 500 when first built. Annually, the hospital attends to about 479,050 patients made up of both out- and inpatients (Biostatistics Unit, 2009). It is the main referral hospital for the Ashanti, Brong Ahafo (BA), northern, upper east, and upper west regions. Statistical record from KATH shows that, about two-thirds of the patients are from the Ashanti Region, with BA and the three northern regions sharing the remaining in a two to one ratio.

St. Martin's Catholic Hospital in Agroyesum is a 100-bed hospital that serves as the referral district hospital for other four health centers in Amansie west district. It is approximately 60 km southwest of Kumasi, the Ashanti regional capital. All hospitalized patients require attendant relatives to provide indirect care and they must provide their own food. Since 1993, the hospital has become the focus for the treatment of Buruli ulcer cases from within and outside the district. This reputation has gained national and international recognition. A US-based foundation, the Humanitarian Aid Relief Team (Provo, UT) has since 1995 been visiting this hospital annually with a team of medical staff to help in the management of patients and also to train local doctors on plastic surgical techniques [15].

The Global Evangelical Mission Hospital (GEMH) is located at Apromase, a village about 10 km southwest of Ejisu, in the Ejisu-Juaben Municipal and also about 12 km southeast of Kumasi. The hospital has thirty-five beds and is well known in the region for its expertise in skin ulcer management; various patients from different facilities have been referred to GEMH from all over the country. The hospital, however, needs additional infrastructure to be capable to accommodate all its patients.

**Ethical Approval**

Ethical clearance was approved by the Kwame Nkrumah University of Science and Technology School of Medical Sciences Ethics Committee, Kumasi, Ghana.

### 2.2. Data Collection and Analysis

Information from BU patients medical records and hospital database from 1994 to 1998 and 2004 to 2007 at three different centers (Global Evangelical Mission Hospital, Apromase; St. Martin's Catholic Hospital, Agroyesum and Komfo Anokye Teaching Hospital, Kumasi) in Ghana by the research team was reviewed to determine the treatment challenges encountered. Information required for the study was retrieved, and the data were recorded, analyzed with Spearman's rank correlation, and displayed in tables and graphs using SPSS version 17.0 (SPSS, Inc., Chicago, IL, USA).

### 2.3. Clinical Diagnosis and Treatment

Patient information was considered for the study when he/she had a laboratory confirmation for BU. Also, BUs for both ulcerated and nonulcerated cases were based on clinical findings and confirmed by any two positives of Ziehl-Neelsen (ZN) test for acid fast bacilli, polymerase chain reaction (PCR), and histopathology. Biopsy samples were taken for all non-ulcerated BU cases for confirmatory analysis. Observations were made by the team to take note of the environment and the lifestyle of the patients. Treatment at the various study sites was based on the current WHO guidelines [16, 17]; BU treatment was surgery (when necessary) combined with chemotherapy (a combination of Rifampicin and Streptomycin for 8 weeks as a first line) and physiotherapy.

## 3. Results

### 3.1. Demographic Features of Patients

**Figure 1:**
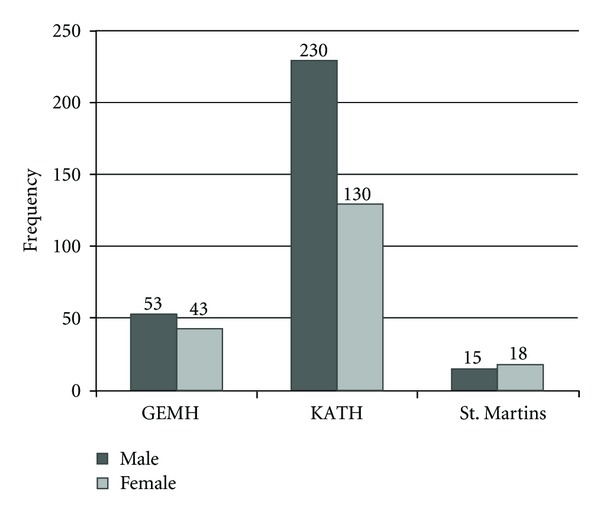
Gender distribution in the various treatment centers. GEMH: Global Evangelical Mission Hospital. KATH: Komfo Anokye Teaching Hospital. St. Martins: St. Martins Catholic Hospital.

**Figure 2:**
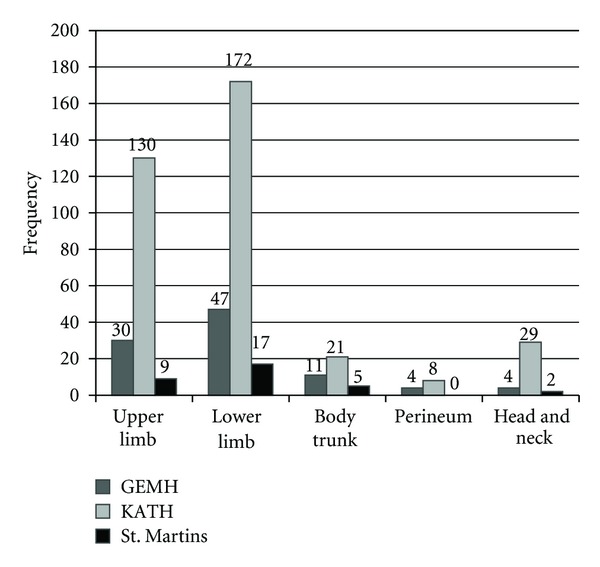
Location of the Buruli ulcer disease in the patients at the various treatment centers.

A total of 489 patients were retrospectively studied from the three treatment centers. A majority (56.90%, *n* = 278) of the patients were children (0–14 years), with a mean age of 12.8 years. KATH recorded the highest (73.60%, *n* = 360) number of patients within the years under review (Figure 1). In terms of location of the infection on the body, lower limbs surpassed all the other parts of the body, with a 48.30% (*n* = 236). Other affected parts of the body included the upper limbs: head and neck region, perineum, and body trunk (Figure 2). 

### 3.2. Challenges in Surgical Treatment

#### 3.2.1. Multiple Surgical Interventions

A total of 30 patients were recruited and treated at GEMH (January–December 2005). Children (0–14 years) constituted 43.4% (*n* = 13) while the adults fall within 33.3% (*n* = 10) with the remaining 23.3% (*n* = 7) being the aged (60+ years). Twenty (66.7%) patients had their lesions on their lower limbs. Also, 23.3% (*n* = 7) patients had their lesions on the upper limbs, 6.3% (*n* = 2) patients had them on the body trunk, and 3.3% (*n* = 1) had them on the perineum. No case was recorded in the head and neck region. 

**Figure 3:**
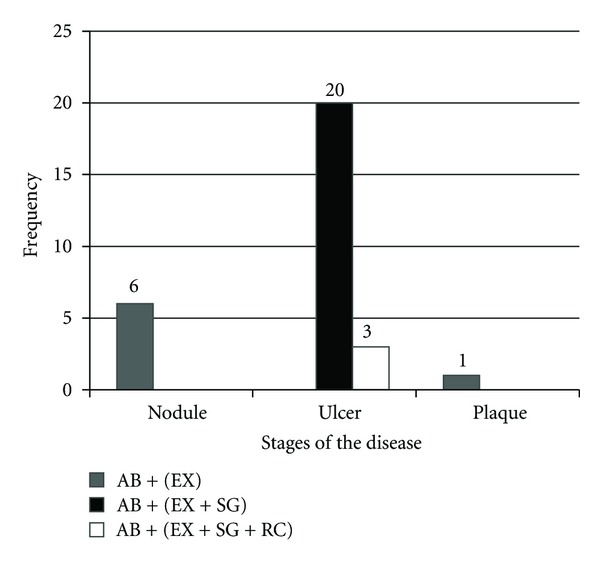
Stages of the disease and treatment received by patients. AB + (EX): antibiotics and excision. AB + (EX + SG): antibiotics, excision, and skin grafting. AB + (EX + SG + RC): antibiotics, excision, skin grafting, and release contracture.

Multiple surgeries were done on the 30 patients except for the nodules (6) and the plaque (1)—that is, seven (7) patients had single surgeries—thus, 23 patients had multiple surgical interventions (giving a total of fifty-six (56) surgical interventions done on these thirty patients). However, none of these 30 patients needed an amputation (Figure 3).

#### 3.2.2. Sequelae of Buruli Ulcer

From 1994 to 1998, a total of 360 BU cases were managed at KATH, Kumasi and two nearby district hospitals (also in the Ashanti Region, Ghana). Sixty-four percent (*n* = 230) were males and 36% (*n* = 130) were females. The majority (66.1%, *n* = 238) were children up to 14 years of age. Out of the 360 BU patients, 56.9% (*n* = 205) were treated by surgical excision followed by split-thickness skin grafting and healed uneventfully. The remaining 43.1% (*n* = 155) had various sequelae (Table 1) and required intensive care and treatment.

### 3.3. Delays in Seeking Medical Treatment

In a study of 33 BU patients in terms of seeking treatment at St. Martin's Catholic Hospital, Agroyesum, from 2006 to 2007, children (0–14 years) constitute 40.0% (*n* = 13). Adults and the aged (60+ years) constitute (33.3%, *n* = 11) and (26.7%, *n* = 9), respectively. Lesions on the lower limbs formed the majority (53.3%, *n* = 17) followed by upper limbs (30.0%, *n* = 10) and body trunk (16.7%, *n* = 6). Most (76.7%, *n* = 23) patients who stayed at home for a longer period, before seeking medical treatment are those who had ulcers (Table 2). 

### 3.4. High Treatment Cost of BUD

A total of 66 patients were treated between July 2004 and June 2006 at the GEMH; 18.2% (*n* = 12) were preulcer cases, 37.9% (*n* = 25) were mild ulcer cases, and 43.9% (*n* = 29) were severe ulcer cases. Males (*n* = 35) outnumbered females (*n* = 31); hospital duration ranged from 8 to 133 days, with a mean duration of 98 days. Age ranged from 1 to 83 with a mean of 15.6. Cost of treatment of BU patients is shown in Table 3. All cost data were expressed in Ghanaian cedis and converted to US$ using an exchange rate of US $1 = *¢* 9,200.00, the average rate during the study period. The disease had been classified into three stages, depending on the number of times a BU patient underwent surgical operations as follows.

**Preulcer**

Preulcer patients had one surgical operation.

**Mild Ulcer**

Mild ulcer patients had one or two surgical operations including skin grafting.

**Severe Ulcer**

Severe ulcer patients had two or more surgical operations including skin grafting.

### 3.5. Socioeconomic Constraints

In this present study, a total of 489 patients (from the three treatment centres) which were mostly residing in rural areas were recruited. Three categories of patients were made up of males and females in the ratio 3 : 2; BU-infected children (0–14 years), adults and the aged (60+ years) were in the ratio 3 : 2 : 0.5, respectively. The working force within the group constitutes 34.2% (*n* = 167) of the total number of patients (Table 4). 

### 3.6. Analysis of Results

#### 3.6.1. Logical Regression Analysis

This was done to determine the significant association between the treatment challenges enumerated and BU. The findings showed that sequelae (*P* = 0.041), delayed treatment (*P* = 0.012), and high treatment cost (*P* = 0.044) were significantly associated with BU management (Table 5).

#### 3.6.2. Spearman's Correlation

The Spearman's analysis was performed under a significance level of 0.05. The correlation is said to be significant when it ranges from +1 to −1. When an analysis is 0 (zero), it means that there is no correlation between the parameters involved. Duration of hospital stay was clearly correlated with the time spent at home prior to admission; Spearman's rank correlation coefficient was 0.72 (95% CI 0.42–0.87).

## 4. Discussion

One of the difficult treatment challenges encountered by members of this present study (although was not statistically significant in the analysis, it is linked to sequelae) was the series of surgeries that some patients had to go through to achieve healing of their lesions, which at times may lead to sequelae. The results of this study shown in Table 2 reveal that twenty-three patients had a total of forty-nine multiple surgical interventions which involved excision, skin grafting, and/or release contracture. The findings of the study revealed various sequelae such as loss of eye, eyelid, nose, and amputations incurred by patients. The sequelae was identified to be contributing to BU management challenges in the endemic areas of Ghana. Also, in the case report of Johnson et al., it was illustrated that series of surgical interventions were performed, and antimycobacterial chemotherapy (Rifampin and Ciprofloxacin) was given for sixty days; however, more than half of the patients had sequelae [18]. Similar surgical interventions were performed in the study of Agbenorku in Ghana in the head and neck region (HNR), for patients who delayed in seeking medical treatment (i.e., patients who reported with BU deformities). Multiple surgeries for treatment and reconstruction of ulcerated eyelids and other facial parts took several days and were very delicate to operate. After the treatment procedures, the MUD had permanently disfigured or partially disabled the patients, since some of the HNR patients were rendered blind. This also demonstrates that BU on the face should be managed judiciously by professionals in the field to enhance proper healing [19].

Delayed in seeking medical care by BU patients resulting in longer stay of patients in the hospital was also ascertained by the study as the strongest factor (*P* = 0.012) in BU treatment challenges, especially for patients who reported with BUD in the ulcerated stage, since the mean hospital duration was ninety-eight days. Most of these patients reported very late for treatment, some even compounded it by using herbal medications, which were additional source of secondary microorganism infections. The 2009 Ghana National BU Report indicates that 64.2% (*n* = 508) of the clinical presentations were ulcers [20], and this had also been confirmed by other BU studies [15, 21–26]. These cases demonstrate the management difficulties of BU in the late ulcerative stage; thus, with ulcer diameters larger than 5 cm surgery was inevitable. The Spearman's analysis indicated that duration of hospital stay was clearly correlated with the time spent at home prior to admission. The severity of sequelae following *Mycobacterium ulcerans* infection causes significant deformities and is related to multiple factors. A major cause is the late presentation of patients to health facilities for treatment. In a survey conducted in Amansie west district in Ghana (most endemic district in Ghana), Asiedu et al. reveal that 60% of the BU patients attributed poverty, beliefs that the treatment does not work, fear of surgery and anesthesia, or superstition and stigma as the causes of delay in seeking medical treatment [25]. The longer a BU patient stays at home, the more difficult to treat him/her and the longer the duration when admitted. Hence, the earlier patients report to appropriate health facilities for treatment could help alleviate the menace of BU sequelae and multiple surgical interventions in BU endemic areas.

Additionally, many of the affected populations do not seek health care because of financial constraints [25], this challenge was buttressed by the regression analysis of this study (*P* = 0.044). In the findings (shown in Table 3), the average total cost of treating preulcer patients, mild ulcer patients, and severe ulcer patients was ($183.24), ($396.07), and ($1,181.14), respectively. Since the mode of the total treatment cost within each category was nil, the direct cost of treating each Buruli ulcer patient varied. These variations were due to the size of the disease and the condition of the patient. The total treatment cost for treating preulcer BU patients was within the range ($140.48–$220.11) inclusive, mild ulcer BU patients ($224.00–$649.70) inclusive, and severe ulcer BU patients ($591.52–$2,508.96) inclusive. The grand total treatment cost of all the three categories of the sixty-six treated Buruli ulcer patients was ($46,191.29). The average total treatment cost for severe ulcer patients was $1,180.65, which was very expensive, and it was 6.43 ($183.25) and 2.98 ($395.58) times the average total treatment cost for preulcer and mild ulcer patients, respectively. The minimum total treatment cost for severe ulcer patients was $591.52, which was 3.93 ($150.48) and 2.64 ($224.00) times the minimum total treatment cost for preulcer and mild ulcer patients respectively. These analyses showed that it was cheaper diagnosing and treating preulcer BU patients in Ghana. 

Asiedu and Etuaful (1998) estimated the total average cost of treating each BU patient at St. Martin's Catholic Hospital as $966.85 in 1994, $706.08 in 1995, and $658.74 in 1996 [27]. The calculated average total treatment cost for all the 66 treated BU patients at GEM hospital from July 2004 to June 2006 was $701.06. Also, Drummond and Butler (2004) reported the average total treatment cost of 26 treated BU patients from 1991 to 1998 in Australian dollars using 1997-98 prices as $6,803.00 for mild cases, $16,525.00 for moderate cases, and $27,681.00 for severe cases. The average total treatment cost of all cases was 14,608.00 Australian dollars [28]. The average total direct cost of treating a BU patient was very high ($701.06), and the total cost of treating all the 66 BU patients was $46,191.28. Out of this amount $34,089.98 was the direct total treatment cost for all severe ulcer patients, $9,908.94 for all mild ulcer patients and $2,192.37 for preulcer patients. If all the 66 treated patients were to be preulcer BU patients, the total direct treatment cost for all of them would have been $12,094.50 and the patients and government would have saved $34,096.78 (73.82% of the total cost).

To alleviate the challenges in BU treatment, personnel from the health sectors especially those in BU endemic areas should educate the communities on appropriate measured as proposed by Agbenorku et al. (2011): the biomedical and psychosocial approach (early detection of the BUD, effective health education, decentralization of antibiotic treatment to the primary health care units in endemic areas, and small-scale grant to the rural patients) [21]. Volunteers from the endemic communities should be well trained and motivated by the Health personnel to assist people in the early detection of the disease and advised them on the benefit of early treatment of BU. When this is implemented, it will increase the number of early reported BU cases, hence reduce the number of late ulcers, simplify the treatment of the BU patient, decrease the hospital admission duration, and minimize the cost involved in the BU treatment.

## 5. Conclusion

In the attempt to identify treatment challenges of BU in endemic areas, delays in seeking appropriate BU treatment were the main factor which resulted in most of the other factors contributing to the challenges in treatment and the socioeconomic status of patients having direct influence on the treatment of BU. A combination of psychosocial and biomedical approach was proposed as holistic method to alleviate the challenges in BU treatment.

## Figures and Tables

**Table 1:** Sequelae of Buruli ulcers.

Sequelae	Number	Percent (%)
Lower extremity contracture	64	41.3
Upper extremity contracture	41	26.5
Amputation within the lower extremities	18	11.6
Amputation within the upper extremities	16	10.3
Loss of eye(s)	5	3.2
Loss of eyelids	6	3.9
Loss of nose (or part of it)	3	1.9
Loss of genitalia (or part of them)	2	1.3

Total	155	100

**Table 2:** Buruli ulcer patients' medical history (*n* = 33).

Stages	Size (cm)	Number	Mean home duration/days	Treatment	Mean hospital stay/days
			(range)		(range)
Ulcers	6–10	6	195 (56–369)	AB + (EX + SG)	87 (41–159)
	10–20	13	264 (117–377)	AB + (EX + SG)^a^	89 (58–139)
				AB + (EX + SG + RC)^b^	
	>20	7	242 (56–369)	AB + (EX + SG)^c^	103 (66–159)
				AB + (EX + SG + RC)^d^	
Nodule	1-2	6	24 (7–56)	AB + (EX)	18 (8–36)
Plaque	3–5	1	28	AB + (EX)	19

^a^11 patients; ^b^2 patient; ^c^4 patients; ^d^3 patients.

Stage: stage of Buruli ulcer at presentation.

Duration: duration of treatment at home prior to admission in days.

Size: size of lesion in centimeters.

Treatment: treatment given:

AB + (EX)—antibiotics and excision

AB + (EX + SG)—antibiotics, excision and skin grafting

AB + (EX + SG + RC)—antibiotics, excision, skin grafting, and release of contracture.

Hospital stay: duration of hospitalization in days.

**Table 3:** Treatment cost distribution for BU patients (in US$).

Cost parameters	Stages					
	Preulcer		Mild ulcer		Severe ulcer	
	Cost in US$	% of total costs	Cost US$	% of total costs	Cost in US$	% of total costs
Card	0.54	0%	0.54	0%	0.54	0%
Documentation	2.17	1%	4.00	1%	11.28	1%
Consultation	8.97	5%	48.22	12%	146.48	12%
Surgery	57.97	32%	55.65	14%	195.65	17%
Anesthesia	53.44	29%	55.22	14%	145.80	12%
Medication	19.65	11%	42.83	11%	116.55	10%
Nursing care	5.03	3%	24.52	6%	75.15	6%
Dressing	4.48	2%	24.11	6%	73.24	6%
Accommodation	17.93	10%	94.87	24%	292.28	25%
Laboratory costs	6.07	3%	13.98	4%	27.13	2%
Average consumable costs	6.98	4%	32.13	8%	97.04	8%

Total costs	183.24 (140.48–220.11)	100%	396.07 (224.00–649.7)	100%	1181.14 (591.52–2508.96)	100%

**Table 4:** Socioeconomic strength of patients in terms of age and gender.

Age	Frequency of patients		Total
	Male	Female
0–14	177	101	278
15–29	45	16	61
30–44	31	35	66
45–59	27	13	40
60–75+	18	26	44

Total	298	191	489

0–14: children; 15–59: working force; 60–75: the aged.

Category I: children, majority of who are pupils.

Category II: working force.

Category III: the aged.

**Table 5:** Regression analysis of treatment challenges.

Treatment challenges	*P* value
Multiple surgery	0.924
Sequelae	0.041**
Delayed treatment	0.012**
Socioeconomic constrain	1.126
High treatment cost	0.044**

**significant level when *P* < 0.05.

## Abbreviations

BU: Buruli ulcer
*MU*: *My* *co* *ba* *ct* *er* *iu* *m* *ulcerans*
WHO: World Health Organization
KATH: Komfo Anokye Teaching Hospital
BA: Brong Ahafo
GEMH: Global Evangelical Mission Hospital
ZN: Ziehl-Neelsen
PCR: Polymerase chain reaction
BUD: Buruli ulcer disease
MUD: *My* *co* *ba* *ct* *er* *iu* *m* *ulcerans* disease
HNR: Head and neck region.
